# The Effect of Protective Mat Thickness on the Upper Limb Strike Force Simulation in Combat Sports and Self Defense

**DOI:** 10.5114/jhk/192131

**Published:** 2024-09-26

**Authors:** Vaclav Beranek, Petr Stastny, Bogdan Bacik, Tomasz Bonkowski, Vit Novacek

**Affiliations:** 1 Department of Rehabilitation Fields, Faculty of Health Care Studies, University of West Bohemia, Pilsen, Czech Republic.; 2 Department of Sports Games, Faculty of Physical Education and Sport, Charles University, Prague, Czech Republic.; 3 Department of Human Motor Behavior, Institute of Sport Sciences, Jerzy Kukuczka Academy of Physical Education in Katowice, Katowice, Poland.; 4 Biomechanical Human Body Models, New Technologies—Research Centre, University of West Bohemia, Pilsen, Czech Republic.; 5 Department of Mechanics, Faculty of Applied Sciences, University of West Bohemia, Pilsen, Czech Republic.

**Keywords:** direct punch, elbow strike, palm strike, biomechanics, mixed martial arts, falling weight impact test

## Abstract

The strike force in combat sports strongly depends on the protective material's mechanical properties and energy absorption capacity. Therefore, this study aimed to estimate the effect of the protective layer thickness and repeated loading on the net force in a falling weight impact test. A falling 8-kg weight dropped from 15 cm, 25 cm, and 50 cm was used to simulate impact peak forces in an upper limb strike. Transfer linear regression functions were identified for three layers of different thickness (1.8 cm, 3.6 cm, and 5.4 cm) between the measured force and undamped force that would be measured if no protective layer was used. A decrease in damping performance under repetitive loading was assessed for the same amount of energy (30 J) absorbed by the specimens. There were 36 specimens examined in 126 tests. When the measuring apparatus was covered with one, two or three layers of Trocellen foam, the undamped force was approximately 2.8, 6.1, and 11.1 times higher, respectively (p < 0.05, R^2^ ≥ 0.95), than the force measured in the kinetic design. This allows researchers to select the number of layers according to the individual needs in terms of safety and injury prevention. A single layer of Trocellen foam used in this study may be insufficient to ensure the safety of athletes in upper limb strike experiments due to possible compaction of the foam structure for deformations exceeding 80% compression and forces exceeding 5 kN, although no injury was previously observed.

## Introduction

The measurements of human strikes span from martial arts (e.g., boxing, kickboxing, karate, and taekwondo) (Culeddu, 2018; Folhes et al., 2023; House and Cowan, 2015), militaries (Vagner et al., 2022), self-defense (Adamec et al., 2021) to biomechanics for legal purposes (Nentwig et al., 2021) wherein the peak force (PF) belongs to the main impact characteristics. Therefore, PF has often been reported for direct strikes (Bingul et al., 2017; Buśko et al., 2016; Halperin et al., 2017a), palm strikes (Neto et al., 2008, 2009, 2012), and elbow strikes (Beranek et al., 2020b). However, the PF measurement must ensure the safety of participants, which requires the use of protective equipment covering the strike contact area (Atha et al., 1985; Dyson et al., 2005; Halperin et al., 2017a; Tong-Iam et al., 2017). This protective barrier may highly influence the measurement results.

The protective equipment such as gloves (Smith et al., 1986) attenuates the contact forces, where the undamped force and acceleration are expected to be higher than the experimentally measured strike force performed with the protective layer (Gupta, 2011). This scenario limits comparisons between different strikes and studies due to the lack of data on the properties of the protective material. The measured force and its peak and impulse are affected by the properties of protective equipment. The peak force can be related to tissue damage (e.g., bone fracture) (Beranek et al., 2020a), and the area under the force-time curve can determine the possibility of concussion during the head impact (Beranek et al., 2020b; Cournoyer and Hoshizaki, 2019; O’Connor et al., 2017; Svoboda et al., 2016). However, a recent review of human strikes (Beranek et al., 2020a) confirmed that comparisons of different combat systems with or without protective equipment are missing in current literature, probably due to the unknow attenuation abilities of protective materials.

Various foam structures are used in many areas in protective gear or for a surface in which sports activity can be safely performed. Sports mats dampening landing are primarily used in gymnastics, high jumps, pole vaults, and combat sports (Mills, 2007). However, there is no product specifically designed for strike force measurements. Thus, a typical solution uses foam on a rigid force plate (Ambroży et al., 2021; Adamec et al., 2021; Beranek et al., 2020b; de Souza and Mattos, 2017; Cholewa et al., 2016; Svoboda et al., 2016). Some authors use moving devices such as pendulums (Atha et al., 1985; Bolander et al., 2009; Chadli et al., 2014; Neto et al., 2012), boxing gloves (Dyson et al., 2005; House and Cowan, 2015; Loturco et al., 2016; Pierce et al., 2006), basketballs (Neto et al., 2008), dummies (Walilko et al., 2005) or moving boxing bags (Bruzas et al., 2018) to ensure the safety of participants (see Supplementary Material 1, 2, 3 and 4). However, the task of direct force detection is best fulfilled by designs with anchored solid platforms (Adamec et al., 2021; Beranek et al., 2020b; Svoboda et al., 2016). In this experimental setting, various foam materials are used to protect the subjects (Beranek et al., 2020b; Halperin et al., 2017b; Loturco et al., 2016), although there are no guidelines for the choice of material and its thickness.

Foam thickness is critical, but only few studies have investigated its effect. With regard to the head impact protection, Lyn and Mills (2001) studied the effect of thickness in polyurethane foams. However, different densities of the samples made it difficult to draw definite conclusions. Tomin and Kmetty (2021) submitted polyethylene foams to falling-weight impact tests. They concluded that a foam thickness limit can be determined for a given load level to avoid excessive compaction of the cells and maximize the shock absorption of the foam. The mechanical properties of closed-cell cross-linked polyethylene foams are influenced mainly by cell structure because higher-density foams have smaller cells and thicker cell walls (Tomin and Kmetty, 2021). Polymer foams are significant in applications where the main task is to protect the user and prevent tissue damage. In these cases, the foam product must absorb the energy during the impact while keeping the generated maximum force below a certain value, e.g., below the limit for facial bone fractures estimated to 5 kN (Beranek et al., 2020b). The damping properties of foam are frequently characterized by the falling weight impact test in which a body with a given geometry and mass is dropped from a given height onto the test specimen while the force is measured (Tomin and Kmetty, 2021, 2022).

Cross-linked polyethylene foam characterized by closed-cell structures is known for its recovery capability after deformation loading (Tomin and Kmetty, 2022). This promotes its repeated use during measuring human strikes, but the foaming process, its cross-linked structure and thickness influence foam’s properties. The grading of sports mats based on such tests is required by various sport-specific standards (*EN 1621-2*, b.r.; *EN 12503-1*, b.r.; *United World Wrestling, Regulations for the licensing of mats*, b.r.). However, the requirements for each sport are different in terms of applicable impact energy, drop height, and falling weight mass, thus it is difficult to compare the properties of products for different applications. Similarly, several test standards describe the falling weight impact test (*ASTM D575-91(2018) - Standard Test Methods for Rubber Properties in Compression*, b.r.; *ASTM D4226-19e1 - Standard Test Methods for Impact Resistance of Rigid Poly(Vinyl Chloride) (PVC) Building Products*, b.r.). However, these standards do not fit with the current application on upper limb strike force measurements and athletes’ protection.

There is a lack of information about the damping properties of cross-linked polymer foam protecting athletes during the upper limb strike force measurement. Various authors use different kinds of protective foam, yet, to our knowledge, they do not assess the damping properties of the materials they use (Beranek et al., 2020a). This makes the comparison among different authors quite difficult. Also, to our knowledge, no product is available on the market that has been specifically designed for such applications. Therefore, the main objective of this study was to design an experiment to simulate a real strike force measurement in athletes using the falling weight impact test to assess the attenuation of the protective foam mat. The secondary objective of this study was to describe the attenuation properties of cross-linked polyethylene foam and to predict real impact force acting without the protective mat during repeated upper limb impact. We hypothesized that it would be possible to predict the value of the measured force at different mat thicknesses as a linear function for one, two, and three layers of the protective mat. The results of this study can be used for the selection of protective mats in strike research and by coaches in combat sports and self-defense to ensure safety during strike training. The concept is based on the numerical prediction of strike force relation to the protective mat.

## Methods

### Experimental Design

The study was set up as a laboratory *in-vitro* experiment under stable loading conditions, where 36 specimens were examined in the total of 126 tests. A falling weight impact test was used to reproduce impact peak forces in the upper limb strikes. The test variables were based on real situation estimates rather than existing standards inconvenient for this particular application. Thus, we considered the previously reported peak force that was up to 10 kN in the upper limb strikes (Beranek et al., 2020b) and the average velocity of the hand during the closed fist and palm strike that exceeded 10 m/s (Beranek et al., 2020a), although higher velocities could occur exceeding 20 m/s (Chiu and Shiang, 1999). The impactor drop heights were selected with regard to the impact velocity, which was 1.7 m/s for 15-cm drop height, 22.1 m/s for 25-cm drop height, and 31.3 m/s for 50-cm drop height. Our preliminary tests demonstrated that these drop heights led to peak forces within the previously observed range (Beranek et al., 2020b). The diameter of the test specimens (74 mm) was chosen according to the corresponding diameter (67.2 mm) of the palm strike contact area (Beranek et al., 2022), which represented the hand contact area with the highest peak force value (Beranek et al., 2020b).

**Figure 1:**
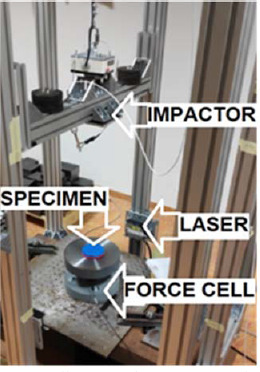
Falling weight impact test device.

The test was performed using an experimental impactor device of high accuracy (Figure 1) (Hynčík et al., 2021; Kottner et al., 2019, 2020) consisting of a highly rigid frame, an impactor, laser distance sensors and a landing area with a load cell. This device is of high accuracy with the measurement error of force of 1–3% and displacement of 2–5% (Hynčík et al., 2021). Each specimen was placed in the center of the rigid support structure. The impactor was released from three different heights: 15 cm, 25 cm and 50 cm. Each specimen was tested three times (three repetitions) with minimum relaxation (till 10 s) allowed for the estimation of material fatigue. Three specimens were tested for all the modalities (three different drop heights, 1–3 layers), thus resulting in 27 specimens and 81 tests. For 50-cm drop height, three additional specimens were tested for all three layers (five repetitions each), thus resulting in nine additional specimens and 45 tests. Therefore, a total of 36 specimens were tested in 126 tests.

#### Experimental Device

An impactor (Figure 1) was attached to a horizontal beam connected to an EATON 1360-CSA (Eaton Corporation PLC, Dublin, Ireland) electromagnet with the maximum holding force of 960 N. The electromagnet was used to release the beam with the impactor in a controlled manner, wherein the impactor and beam together weighed 8 kg, and its contact area included a rectangular head of 12 cm × 14 cm. The impactor was placed at a given height above the specimen on a rigid support mounted on a 50-kN KISTLER 9351B (Kistler, Winterthur, Switzerland) load cell for the weight impact test. The load cell was mounted on a highly rigid support weighing 500 kg into a sliding mechanism with friction-reducing Teflon inserts, which allowed the impactor's horizontal beam to move along two vertical beams. The total dimensions of the construction were 1.4-m wide, 1.4-m long and 2 m of height. Each vertical beam was equipped with a MICRO-EPSILON optoNCDT (Micro-Epsilon Messtechnik GmbH, Ortenburg, Germany) laser distance sensor measuring the distance between each end of the horizontal beam and the plane on which the specimen was placed.

### Experimental Material

A Trocellen (Trocellen Italia S.p.a, Treviso, Italy) cross-linked closed-cell polyethylene foam mat of 18 mm in thickness was sampled into 74 mm in diameter circular pieces. The mat model was the Tatami Karate WKF certified by the World Karate Federation. Two and three pieces were assembled to form specimens with two and three layers, respectively. Double-face tape was used to attach two and/or three pieces together. Thus, specimens consisting of one (18 mm), two (36 mm) and three (54 mm) layers were obtained.

### Optical Microscope Observations

Additional optical microscope observations were performed to estimate the compaction level of a foam specimen under compression. An Olympus DSX100 (Orinpasu Kabushiki-kaisha, Shinjuku, Tokyo, Japan) opto-digital microscope was used. A block of foam was cut. Its free height was measured using a caliper with 0.02-mm resolution. The specimen was placed freely under the microscope so that both red and blue layers of the foam would be visible. The pictures of the specimen were taken at 1.5 × and 6.0 × magnification. Then the specimen was placed between two metal plates and gradually compressed to 80%, 60%, 40% and 20% of its initial height. For each level of deformation, two pictures of the specimen were taken at 1.5 × and 6.0 × magnification. At 80% deformation (20% of the initial height), the excessive bulge of the specimen occurred. The specimen was adjusted and the bulge was cut with a razor blade.

### Data Postprocessing

**Figure 2:**
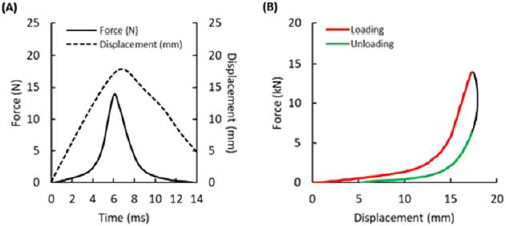
Force time and force-displacement data from a load cell during the weight impact test. (A) Illustration of a typical displacement-time and force-time curve. (B) Illustration of a typical displacement-force curve. Loading (ascending) and unloading (descending) portions are highlighted in red and green, respectively. Black parts of the curve were discarded.

MATLAB® R2019b (MathWorks®, Inc., Natick, MA, USA) was used to post process the recorded data. Impactor displacement and force were post processed as functions of time, and displacement was computed as an average of both laser distance sensors. In addition, only the part of the displacement-force curve corresponding to compression and subsequent release of the specimens was post processed; the remaining data were discarded (Figure 2A). The displacement-force curve was split into the loading (ascending) and unloading (descending) portions. The loading part was taken from the beginning of the curve to the maximum force. In the remaining part of the curve, data points with compression values higher than the one at which the maximum force occurred were discarded. Thus, the unloading (descending) part was obtained. The process is illustrated in Figure 2B.

For each drop height and repetition, peak force values (F) and corresponding displacement (x) (compression of the specimen) were identified and fitted with an exponential function (Hyncik et al., 2021):


F(x)=a⋅exp(bx)(1)

The extrapolation of the force to zero displacement resulted in F(0) = a. This result may be interpreted as an estimate of force that the load cell would measure if there was no protective mat specimen. This estimate is called the undamped force in the following sections and was used to describe the relationship between the measured peak force and undamped force (Hyncik et al., 2021). The data points that were obtained in this process were fitted using a linear function with zero intercepts for one, two and three layers. The area under the loading (ascending) portion of the displacement-force curve corresponded to the total energy (E) absorbed by the specimen, while the area under the unloading (descending) portion of the displacement-force curve corresponded to the elastic energy (E_E_) that was subsequently released from the mat, which contributed to the rebound of the impactor. The difference:


$${\text{E}}_{\text{p}}=\text{E}-{\text{E}}_{\text{E}}\left(2\right)$$

was the energy E_P_ of the plastic deformation of the mat. This energy was not released upon unloading. Instead, it was dissipated by the specimen and expanded to its permanent deformation (Kolupaeva and Semenov, 2015). The reference energy (E_ref_) was computed as the energy absorbed by the specimens when the force threshold of 10 kN was reached in the falling weight impact tests with one layer, 50-cm drop height and one repetition. Force corresponding to the energy E_ref_ was assessed in all the remaining tests with 50-cm drop height.

### Statistical Analysis

All statistical procedures were done in MATLAB® R2019b (MathWorks®, Inc., Natick, MA, USA) with the level of significance set at 0.05 (power alfa). The data normality in each set of measures (different falling heights and layers) was checked with the Shapiro-Wilk test. Since all data were normally distributed, parametric statistics were applied.

The relationship between the measured peak force and undamped force was assessed using data derived from equation (1) for each number of layers separately, where linear regression (function fitlm) was used with exponential curve fitting (function fit). The data points that were obtained in this process were fitted using a linear function with zero intercepts for one, two and three layers. Thus, three linear transfer functions were obtained, with the slope being a factor by which the measured force needed to be multiplied to obtain the undamped force. Linear regression was used at a 95% confidence interval (95% CI), and the coefficient of determination R^2^ was provided.

The effect of repeated loading was analyzed from mean and standard deviation of the force corresponding to the energy absorption E_ref_ (Equation 1) for all three layers and five repetitions. Differences were compared by one-way ANOVA for repeated measures followed by a left tail post hoc *t* test to compare force/repetition differences. Finally, the relative force increase in repetition 1 was assessed for all three layers, which showed the effect of repeated loading on force with the same amount of energy absorbed by specimens.

## Results

### A Falling Weight Impact Test

**Figure 3:**
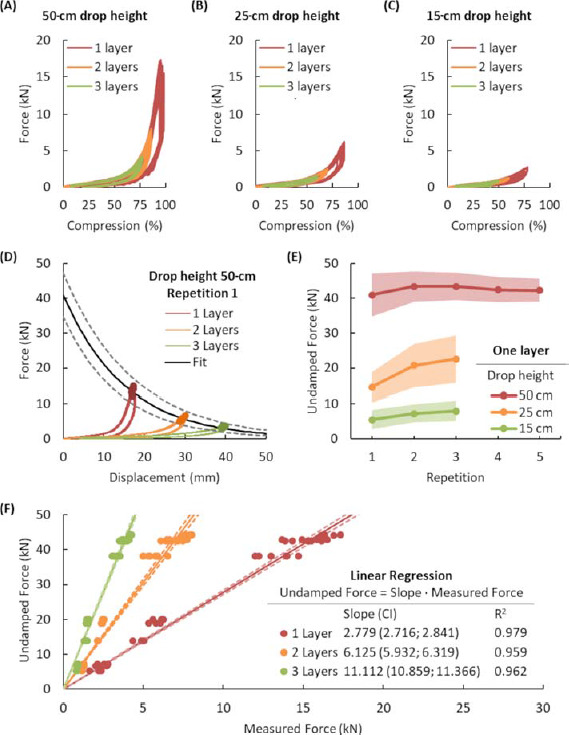
Results of the falling weight impact test for each testing condition and linear regressions for each protective mat thickness. Force-compression curves for 1, 2, and 3 layers for (A) 50-cm drop height, (B) 25-cm drop height, and (C) 15-cm drop height. (D) Results of the falling weight impact test for 50-cm drop height and repetition 1. (E) Undamped force for all three drop heights, three layers, and three or five consecutive repetitions (light color areas represent 95% CI). (F) Results of the linear regression (linear transfer function). Peak force values are highlighted by circular markers. Layer 1 = 1.8-cm thickness, 2 layers = 3.6-cm thickness and 3 layers = 5.4-cm thickness.

Falling weight impact force-compression curves are presented in Figure 3(A–C). They are shown for all three layers and all three drop heights. In Figure 3(A) one can notice that the single layer became compacted at about 75% of compression and force exceeding 5 kN. With regard to two and three layers, it seems that the foam mat did not reach compaction in the 50-cm drop height falling weight experiment.

Falling weight impact test peak force data were fitted using the exponential function (1) that is frequently applied as a model of decay or damping (Figure 3D). Extrapolation to 0-mm displacement may be interpreted as an estimate of force that the load cell would measure if there was no protective mat specimen. The undamped force was estimated in the same manner for all of the test modalities: three drop heights, three layers and up to five repetitions (Figure 3E). The increasing trend of these curves suggested a decreasing capacity of the mat specimens to absorb the impact energy and to attenuate the force. The undamped force was further expressed as a function of the measured peak force, wherein the slope was a factor by which the measured force needed to be multiplied to obtain the undamped force (Figure 3F). In this linear transfer function with zero intercepts, the coefficient of determination at the 95% CI showed very high predictability (R ≥ 0.95, Figure 3F). For one layer, the undamped force was approximately 2.8 times higher than the measured force and 6.1 and 11.1 times higher than the measured force for two and three layers of protection, respectively. Moreover, two and three layers were able to attenuate the force 2.2 times and 4 times more compared to a single layer.

### Effect of Repeated Loading

**Figure 4:**
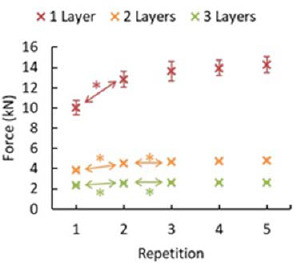
Force vs. repetition differences during the repeated loading test. The force (mean ± standard deviation) reached at 30 J of energy absorbed by the specimens at 50-cm drop height in the falling weight impact test for all the repetitions and for all of the three layers. Layer 1 = 1.8-cm thickness, 2 layers = 3.6-cm thickness, and 3 layers = 5.4-cm thickness. The symbol * denotes a significantly higher value in repetition n compared to repetition n−1.

Repeated loading reduced the energy absorption capacity of the mat as it accumulated plastic deformation. The loading portion of the force-displacement curve was integrated to obtain the total energy E absorbed by the specimen as a function of displacement. Falling weight impact tests with one layer, 50-cm drop height and one repetition were considered the reference tests. The energy absorbed by specimens was assessed when a force threshold of 10 kN was reached. The energy corresponding to this force threshold was 30 J. This value was in agreement with the falling weight experiments using cylindrical impactors of 50- and 100-mm diameter dropped from 40 cm resulting in the impact energy of 21.98 J and 39.90 J, respectively (Tomin and Kmetty, 2021). Additionally, force corresponding to the energy of 30 J was observed in all the remaining tests with 50-cm drop height (Figure 4, Table 1). According to the ANOVAs (*p* < 0.05) and one-sided post hoc *t* tests, the second repetition showed higher force than the first one for one, two and three layers. The force in the third repetition was significantly higher than that in the second repetition only in two and three layers (Figure 4, Table 1).

**Table 1:** The force (mean ± standard deviation [STD]) reached at 30 J of energy absorbed by the specimens at 50-cm drop height in the falling weight impact test. The force increase shows the increase in force concerning repetition 1 for a given number of layers. A *p*-value < 0.05 shows a significant difference between repetition n and repetition n−1. Layer 1 = 1.8-cm thickness F = 22.70, η^2^ = 0.872 , 2 layers = 3.6-cm thickness F = 48.58, η^2^ = 0.911, and 3 layers = 5.4-cm thickness F = 77.33, η2 = 0.942.

Layers (n)	Repetitions	Force (N) (Mean ± STD)			Force Increase (%)	*p* value
1	1	10017	±	739	–	–
	2	12867	±	781	28	< 0.001*
	3	13642	±	946	36	0.076
	4	13967	±	775	39	0.313
	5	14250	±	786	42	0.340
2	1	3858	±	124	–	–
	2	4525	±	121	17	< 0.001*
	3	4683	±	133	21	0.028*
	4	4767	±	104	24	0.189
	5	4833	±	161	25	0.290
3	1	2342	±	20	–	–
	2	2558	±	38	9	< 0.001*
	3	2608	±	38	11	0.022*
	4	2600	±	0	11	0.639
	5	2617	±	29	12	0.187

* significantly different at p < 0.05

#### Optical Microscope Observations

**Figure 5:**
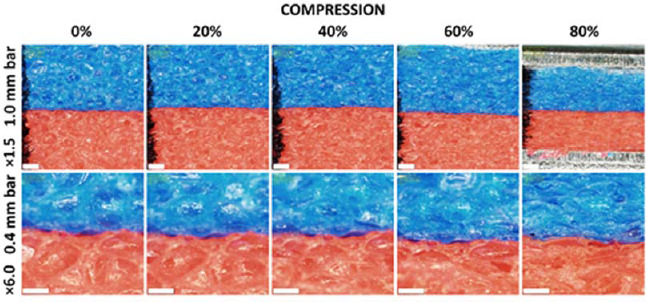
Optical microscope observations of the Trocellen foam specimen at 0, 20, 40, 60 and 80% compression. The same region of interest is shown for 0% to 60% deformation. Excessive bulge occurred at 80% of deformation and it was cut with a razor blade. Thus, the images show different structures at 80% of compression. The upper row shows a foam structure with 1.5× magnification. The white bar indicates a 1.0-mm scale. A black marker was used to create a vertical line on the left side to help place the specimen. The lower row shows a foam structure with 6.0× magnification. The white bar indicates a 0.4-mm scale.

We also performed microscope observations of the foam at different levels of compression. Although progressive closing of cells occurred due to the compression, it seems that the compaction was not complete at 80% deformation. Some cells remained open although highly deformed (Figure 5).

## Discussion

This study aimed to estimate the effect of the protective layer thickness and repeated loading on the net force in a falling weight impact test which was used to evaluate the force attenuation capacity of the Trocellen protective mat. We can confirm our hypothesis, since linear transfer function with very high predictability was identified for one, two, and three layers of the protective mat, thus providing force that would be measured if there was no protective layer at all. The undamped force was approximately 2.8, 6.1, and 11.1 times higher than the measured force for one, two and three layers of protection, respectively. These data suggest that previous findings reporting the peak net force with protective equipment (Adamec et al., 2021; Bolander et al., 2009; Halperin et al., 2017b) have highly underestimated the actual force interaction, which takes into account action without the use of these experimental protective tools.

Repetitive loading with minimum relaxation time decreases protection capacity. This degradation in energy absorption capacity was mostly apparent in the first two repetitions (Table 1) and became stable starting from the third repetition. The highest force increase due to repeated loading was observed in one layer, and the lowest force increase was observed in three layers of protection.

This observation is of significance especially when athletes are assessed for repeated strikes (Bruzas et al., 2018) with a minimum rest interval in between (Halperin et al., 2017a, 2017b; Pierce et al., 2006). Therefore, we can recommend discarding the first one or two attempts considering them a warm-up before the experiment starts due to providing low performance of strikes in terms of the generated force. Another option is to change the protective mat after each impact. However, this would be costly and time-consuming. The designers of experiments should include safety margins and consider both protective material thickness and repeated loading effects. They might also consider using an additional layer to ensure safety of the subjects.

The measurement of impact force in combat sports is especially important in the context of injury prevention, especially for the head. The head is a monitored segment in all contact sports (Abrahams et al., 2014; Cournoyer and Hoshizaki, 2019) especially in those activities where the head is the most common target (Follmer et al., 2019; Tong-Iam et al., 2017). Head injuries are predominant in mixed martial arts (MMA) (Lockwood et al., 2018; Miarka et al., 2019) wherein athletes can use almost any strike at its highest intensity (Miarka et al., 2019). In a previous study (Beranek et al., 2020b), the impact force of three strikes commonly used in the ground and pound positions in the MMA system was measured with a force plate covered with a Trocellen protective mat. The average forces were 2.9 kN for a direct punch, 4.1 kN for a palm strike and 3.8 kN for an elbow strike. If one were to use our regression method, the corresponding undamped forces would be 8.1 kN, 11.4 kN and 10.6 kN for direct, palm and elbow strikes, respectively. Thus, there would be a high possibility of succeeding critical values of 5.1 kN under real combat conditions (Beranek et al., 2020a).

The diameter (74 mm) of the specimens was selected for the contact area estimated in a palm strike technique that was expected to exhibit the maximum contact area compared to direct and elbow strikes. A value of 14.13 cm^2^ was reported for the contact area of the gjaku cuki technique (Droščák, 2017), which corresponds to an equivalent diameter of 42 mm. This technique (when correctly executed) is known to use only a small part of the hand. Furthermore, 6.5 cm^2^ was reported for the contact area of the Taekwondo punch (Svoboda et al., 2016) which corresponds to an equivalent diameter of 29 mm. Those authors hypothesized that a properly performed punch would use only the knuckles of the index and middle fingers. The diameter of specimens was close to the recently reported value of 67.2 mm (Beranek et al., 2022). However, the dimensions of the imprints in different strike techniques were measured against a rigid glass surface. The contact area with the mat might be slightly larger due to its deformation under the action of the limb.

This study presents some possible limitations in the falling weight experiment. The specimens were compressed by the impactor larger and wider than the specimens, while in the upper limb strike, the athlete hits the mat covering the entire force plate with dimensions of 60 cm by 40 cm (Beranek et al., 2020b). The mat is compressed under the limb, however, in contrast to the falling weight experiment, it is free to bulge around the limb. We also neglected the damping effect of the double-face tape. Other types of methods for joining the layers of the mat (for example, by gluing the pieces together to form a multilayer specimen) could change the chemical structure of the mat and influence the results. The deformation increase after unloading (apparent in Figure 2E) may be attributed to material creep. Viscous effects were neglected in this study, and only elastoplastic material behavior was considered. Moreover, the maximum deformation frequently approached almost 100%. This result may be attributed to the measurement inaccuracy having two possible origins. The first possible source of error resides in the manual setup of the initial impactor height. The second one is related to the slightly uneven movement of both sides of the beam carrying the impactor. The compressive deformation experienced by the specimen was likely nonhomogeneous. Nevertheless, compared to statistical variability in the specimens, this inaccuracy had only a negligible effect on the results.

From force-compression curves, it seems that foam compaction occurred in one layer of the protective mat in the 50-cm drop height experiment. The porous structure of the mat collapsed and the mat started to behave as a plain material. The compaction seems to have started at around 75% of deformation for forces exceeding 5 kN. Above this threshold, one layer experienced a different deformation mode than two and three layers. Microscope observations confirmed compaction of the foam at 80% compression although it was not complete, and some cells remained open. The fitted coefficients of the transfer function might be limited to the test cases in this study, namely to the particular model of the Trocellen foam, which also is a limitation of this study.

Athletes might be at risk when performing upper limb strikes exceeding 5 kN peak force with only a single layer of the Trocellen protective mat tested in this paper. However, no injury was observed in previously performed experiments with peak forces reaching up to 10 kN (Beranek et al., 2020b). It may be recommended using an additional layer of protection to increase safety and avoid possible injury due to the compaction of a single layer. Another safety factor is the necessary support of the foam, where a higher number of layers can also mean unwanted movement of the foam under impact. It depends on the specific requirements and the profile of the tested probands and their experience with a specific type of a strike. Compaction of the protective foam should not start below 10 kN peak force to ensure the polymer's energy absorption capacity has not been reduced or compromised due to the collapsing cell structure. The sudden increase in force in the so-called densification zone occurs because a further compression of the air between the closed cells leads to an increase in pressure. Further deformation leads to the collapse of the cells; their opposite walls get into contact and the foam starts to behave as a plain material with reduced energy absorption capacity. These results could serve as a guideline for researchers performing upper limb strike force measurements and experiments. They may also help manufacturers in designing products for this specific application.

## Conclusions

This study enabled the calculation of the undamped force in the kinetic measurement design using the selected damping foam mat. The undamped force was approximately 2.8, 6.1 and 11.1 times higher than the measured force for one, two and three layers of protection, respectively. This allows researchers and coaches to select the number of layers according to the needs of subjects in terms of safety and injury prevention. Repeated loading significantly affects only first two loading cycles in one layer and only first loading cycle in two and three layers of Trocellen foam.

## Figures and Tables

**Supplementary material 1:** Overview of kinematic and kinetic measurement designs of strikes by the upper limb. No study reported the impact foam attenuation.

Author(s), year	Measuring device for impact force of striking by the upper limb	Rigid/movable target	Direct/Indirect force design	Impact plate cover
Beranek et al., 2020	Kistler 9286 B, Kistler Inc instrumente, GmbH, Winterthur, Switzerland	rigid	direct	polyethylen Trocelen
de Souza et al., 2017	four strain gauges on the punching board, load cell ZX 5T, Alfa InstrumentosEletronicos, Ltda	movable	direct	makiwara m wooden plate rope (Sisal)
Chiu et al., 1999	accelerometer (3 axial)	movable	indirect	20-cm protective pad material
Bolander et al., 2009	accelerometers model 7264D a load cellmodel 7120 Syscon Inc	movable	direct	pendulum system in experimental design, a foam pad with a thin ABS plastic covering
Neto et al., 2008	a high-speed digital imaging system (MotionScope@ jnkPCI, Model 8000s, Red Lake, USA) adjusted to 1000 Hz	movable	indirect	composite leather basketball 0.597 kg
Neto et al., 2009	load cell (model 7120 Syscon Inc.) a Tekscan high-speed pressure sensor (Model 9500)	movable	direct	pendulum system in experimental design, a protective pad
Neto et al., 2012	load cell (model 7120 Syscon Inc.)	movable	direct	pendulum system in experimental design, a foam pad with a thin ABS plastic covering
Walilko et al., 2005	aeven Endevco (San Juan Capistrano, CA) 72642000 Accelerometers a six-axis upper neck load cell (Denton ATD, Rochester Hills, MI)	movable	indirect	hybrid 3 Dummy with boxing helmet, standard boxing gloves
Loturco et al., 2016	a force platform with custom-designed software (Accu-Power; AMTI, Graz, Austria),	rigid	direct	protective pad Body shield Bad Boy, San Diego, CA, USA, boxing gloves
Chadli et al., 2014	strain gauges, accelerometers	movable	direct	pendulum system, elastic element for absorb incident punch energy, boxing gloves
Busko et al., 2016	accelerometer (The new boxing Training simulator system BTS-4AP-2K)	movable	indirect	dynamometric punching bag
Bružas et al., 2015	kiktest-100 (a dynamometer and a sensing element to register the impulse of blows)	movable	direct	standard boxing bag
Halperin et al., 2017a	a load cell Two phototransistor infrared LED lightgates (Vishay)	rigid	direct	metal plate covered by a large foam pad wrapped by leather envelope, boxing gloves
Halperin et al., 2017b	a load cell Two phototransistor infrared LED lightgates (Vishay)	rigid	direct	metal plate covered by a large foam pad wrapped by leather envelope, boxing gloves
House et al., 2015	f scan force senzor Tekscan, Inc. Boston, MA	movable	direct	punching bag, boxing gloves
Dyson et al., 2005	dynamometr Kistler 330 Hz	movable	direct	boxing dynamometer, boxing gloves
Pierce et al., 2016	force sensor inside gloves	monitoring during boxers’ competitive matches in real time	direct	boxing gloves
Svoboda et al., 2015	dynamometric plate Kistler 9281	rigid	direct	foam pad
Atha et al., 1985	accelerometer	movable	indirect	pendulum system with a plate 0.8 kg with felt and leather to a thickness and yield approximating that of the human face, boxing gloves
Čepulénas et al., 2011	kick test	movable	direct	boxing dynamometer, boxing gloves
Adamec et al., 2021	Kistler force plate 9286B	rigid	direct	punching pad 40 x 20 x 11
Lenetsky et al., 2018	accelerometer	movable	indirect	boxing bag

**Supplementary material 2:** Overview of kinematic-only measurement designs of strikes by the upper limb. No study reported the impact foam attenuation.

Author(s), year	Measuring device for impact force of striking by the upper limb	Rigid/movable target	Direct/Indirect force design	Impact plate cover
Smith, 1983	camera	movable	indirect	heavy bag
Cesari et al., 2007	two pairs of photocells	movable	indirect	boxing bag from wood covered with material of stones and sand with total weight of 25 kg, medium soft material Polyurethane foam.
Gianino et al., 2010	digital movie camera	only movement	indirect	
Daniel et al., 2014	the Quintic information system (for the motion analysis and evaluation)	only movement	indirect	
Rinaldi et al., 2018	340-Hz optoelectronic motion analysis system SMART-DX6000 System, BTS, Milan, Italy 8 infrared cameras	movable	indirect	punching bag, 138 cm height, 130 kg mass, fix on the ground
Tong-Iam et al., 2017	qualisys Oqus 7, Qualisys AB, Sweden	movable	indirect	punching ball 5 kg
Cheraghi et al., 2014	two camera KODAK	rigid	indirect	foamed wood target 30 x 50 x 5 cm, boxing gloves
Bingul et al., 2017	eight synchronised high-speed cameras (Oqus 7+)	movable	indirect	sand bag
Kimm et al., 2015	accelerometer	only movement	indirect	boxing gloves

**Supplementary material 3:** Overview of kinetic measurement designs of strikes by the upper limb. No study reported the impact foam attenuation.

Author(s), year	Measuring device for impact force of striking by the upper limb	Rigid/movable target	Direct/Indirect force design	Impact plate cover
Falco et al., 2008	Body heavy dense foam padded with force plate, pressure senzors	rigid	direct	foam padded
Estevan et al., 2011	Body heavy dense foam padded with force plate, pressure senzors	rigid	direct	foam padded
Vagner et al., 2018	Kistler 9281	rigid	direct	field shoes designed for the Armed foam
Vagner et al., 2021	Kistler 9281	rigid	direct	Industrial strength vinyl tatami 200 mm
Pedzich et al., 2006	Force plate	rigid	direct	absorption layer

**Supplementary material 4:** Overview of kinematic measurement designs of strikes by the upper limb. No study reported the impact foam attenuation.

Author(s), year	Measuring device for impact force of striking by the upper limb	Rigid/movable target	Direct/Indirect force design	Impact plate cover
Vasik and Shan, 2015	Smart-D system, BTS S.p.A., Italy	rigid	indirect	wooden plate
Vasik, 2011	Smart-D, BTS S.p.A., complex movement analysis.	rigid	indirect	L-stance guarding block
Preuschl et al., 2016	High speed cameras	movable	indirect	kicking pad (PR1614 Hand Mit, Daedo, Barcelona, Spain
Chang et al., 2021	Vicon camera system	not reported	indirect	no report
Moreira et al., 2015	Cameras	movable	indirect	handheld target Daedo®, Barcelona, ESP
Gavagan et al., 2017	Camera system Qualisys AB, Gothenburg, Sweden	not reported	indirect	target pad
